# Therapeutical and Pharmaceutical Notes

**Published:** 1900-07

**Authors:** W. J. Martin

**Affiliations:** Kankakee, Ill.


### THERAPEUTICAL AND PHARMACEUTICAL NOTES.

UNDER THE DIRECTION OF W. J. MARTIN, M.D.C.,
KANKAKEE, ILL.

ZEROFORM IN NEURECTOMY. During the past year we have used this drug quite extensively in the performance of the various forms of plantar neurectomies, and can confidently recommend its use to the members of the profession as a reliable antiseptic and germicide.

Zeroform is a yellow powder, containing 50 per cent. of oxide of bismuth in chemical combination with 50 per cent. of tribromophenol. It is nearly odorless, and is more economical to use in practice than iodoform, because in equal volume with iodoform its weight is about half that of the latter drug. Hence the price of zeroform at 60 cents per ounce is about as cheap as iodoform at 40 cents per ounce.

Our method of using zeroform in neurectomy is as follows: The patient is placed on an operating table, the limbs are thoroughly washed in a ½ per cent. solution of formalin, the hair over the seat of operation is removed by shaving with a razor, and the parts again well washed. A 10 per cent. solution of cocaine or beta-eucaine is then injected at the superior head of the metacarpal bone, and directly over the plantar nerve. We prefer this site for the injection because the field of operation is not obscured by the infiltration of the tissues with the fluid. We also found by experiment, that the anæsthetic action secured was as complete as when the injection is made directly over the site of operation. After the application of an Esmarch elastic bandage to the limb, the nerve is resected in the usual manner. After removal of the section of the nerve, the edges of the wound are brought into apposition with a fine formalized-catgut ligature. Any oozing of blood is soaked up with small pieces of absorbent cotton, the parts well covered with zeroform, the limb covered with a layer of absorbent cotton, and a wet formalized bandage applied over all. This dressing is not changed for five or six days, when the wounds will have united by first intention. Since employing zeroform in this operation, the results have been in every case most gratifying.

A Plaster of Oxide of Zinc. Unna suggests the following formula: Linseed-oil, 5 drachms; lime-water, 5 drachms; oxide of zinc, 1 ounce; prepared chalk, 1 ounce. This makes a paste, in which various other drugs may be employed for diseased conditions of the skin. The following substances, however, should not be mixed with it: Acetic acid, salicylic acid, pyrogallol, phenol, resorcin, or ichthyol. On the other hand, the following medicaments may be added to this paste for application in certain cases of skin-diseases with advantage: Corrosive sublimate up to the strength of 10 per cent.; chrysarobin, 10 per cent.; balsam of Peru, 10 per cent.; oil of cade, 5 per cent.; tar, 10 per cent.; extract of cannabis indica, 5 per cent.; extract of opium, 6 per cent.; menthol, 2 per cent.; oil of almonds, 10 per cent.; and cocaine, from $\frac{1}{2}$ to 1 per cent.—*Therapeutic Gazette.*

The above paste will, without doubt, prove of value to the veterinarian in the treatment of many of the troublesome skin affections so often met with in practice, more especially those found on the legs of horses, such as eczema, erythema, etc.—*W. J. M.*

Treatment of Basedow's Disease by the Milk of Thyroidectomized Goats. Lanz records the results which he has obtained in the treatment of Basedow's disease with the milk of goats from which the thyroid gland had been removed.

The idea of the symptoms of Basedow's disease is that they are due to hypersecretion of the thyroid gland, and this may be to a certain extent modified if the thyroid gland is removed. It occurred to Lanz that as this latter operation could not be performed, it might be possible to influence the system of the patient by the use of milk derived from goats which had been deprived of this gland, at least in part.

The milk was administered to three cases. In the first the treatment was followed in fifteen days by a diminution of the frequency of the pulse, disappearance of the headache and insomnia, improvement of the appetite, and notably diminution in the size of the goitre. In the second patient, after a period of nine weeks, there was noticed a diminution in the frequency of the pulse, improvement in the appetite and sleep, diminution of the tremor, and a general systemic improvement. Trousseau's sign disappeared. In the third case, at the end of eight days, there was a diminution of the nervous excitation and exophthalmia.

These patients took from two to three glasses of milk per day.—*Ibid.*

Tannoform in Diarrhœa. M. Lemberger has recently shown (*Thierarztliches Centralblatt*) the effect of tannoform in obstinate cases of diarrhœa. In the first place he used it in the case of a heifer affected with an acute intestinal catarrh upon which ordinary astringent remedies had no effect. He gave it in doses of ten grammes in one litre of chamomile-tea three times a day. In five days the appetite had returned, and the animal was on the high road to recovery. Another case was treated in the same way with an equally satisfactory result. Lemberger is particularly emphatic in pointing out that though the amount of the drug used was considerable (ninety grammes in forty-eight hours), the gastric function was not in any way interfered with. This he believes to be explicable by the insolubility of tannoform in acid liquids. It is, therefore, only decomposed when it reaches the intestines, where tannin and formaldehyde are produced, and exert their astringent and antiseptic actions.—*Veterinary Journal.*

Aside from its virtues as an intestinal antiseptic, tannoform has of late been quite extensively used by dermatologists in the treatment of various skin diseases; and also in suppurating sores and ulcers. It is employed both in the form of a dusting powder and ointment, and is highly spoken of as an efficient agent in these affections.—*W. J. M.*

Acarus-mange in Dogs. Veterinary surgeon Paust recommends the following treatment as having been successful in a case of a terrier which had been declared incurable. Morning and evening vigorous scrubbing in warm water and a solution of green soap, then brushing and syringing with a solution of creolin, then syringing with and dipping the whole body for several minutes into a strong decoction of tobacco. After drying, the following ointment is used: Sulph. precipit., zinc sulph., each, 1 gramme; lard, 8 grammes.

Bedding to be renewed daily, fresh clean straw below and soft warm cloths on top. No harm need come to the person who performs this work. More than once I thought the dog was going to die through sheer weakness. Wine and beef-tea were forcibly administered to the animal, as all appetite had disappeared. After awhile the above treatment could be suspended every second day, and at the end of a little more than a week it was stopped, and the cure was complete. Dogs exhibit symptoms of great pain after rubbing with the ointment, and run about howling; a period of great weakness follows. Strict attention must be paid to the method of procedure, and no time or trouble ought to be spared if a cure is to be complete.—(*Berliner Thierarzt Woch.*) *Veterinary Journal.*

